# Extracellular Histones Induce Chemokine Production in Whole Blood *Ex Vivo* and Leukocyte Recruitment *In Vivo*

**DOI:** 10.1371/journal.ppat.1005319

**Published:** 2015-12-08

**Authors:** Johannes Westman, Praveen Papareddy, Madelene W. Dahlgren, Bhavya Chakrakodi, Anna Norrby-Teglund, Emanuel Smeds, Adam Linder, Matthias Mörgelin, Bengt Johansson-Lindbom, Arne Egesten, Heiko Herwald

**Affiliations:** 1 Department of Clinical Sciences, Division of Infection Medicine, Biomedical Center, Lund, Sweden; 2 Department of Experimental Medical Science, Adaptive Immunity, Biomedical Center, Lund, Sweden; 3 Department of Medicine, Center for Infectious Medicine, Karolinska Institutet, Karolinska University Hospital Huddinge, Stockholm, Sweden; 4 Department of Clinical Sciences, Respiratory Medicine & Allergy, Biomedical Center, Lund, Sweden; University of California, Davis, UNITED STATES

## Abstract

The innate immune system relies to a great deal on the interaction of pattern recognition receptors with pathogen- or damage-associated molecular pattern molecules. Extracellular histones belong to the latter group and their release has been described to contribute to the induction of systemic inflammatory reactions. However, little is known about their functions in the early immune response to an invading pathogen. Here we show that extracellular histones specifically target monocytes in human blood and this evokes the mobilization of the chemotactic chemokines CXCL9 and CXCL10 from these cells. The chemokine induction involves the toll-like receptor 4/myeloid differentiation factor 2 complex on monocytes, and is under the control of interferon-γ. Consequently, subcutaneous challenge with extracellular histones results in elevated levels of CXCL10 in a murine air pouch model and an influx of leukocytes to the site of injection in a TLR4 dependent manner. When analyzing tissue biopsies from patients with necrotizing fasciitis caused by *Streptococcus pyogenes*, extracellular histone H4 and CXCL10 are immunostained in necrotic, but not healthy tissue. Collectively, these results show for the first time that extracellular histones have an important function as chemoattractants as their local release triggers the recruitment of immune cells to the site of infection.

## Author Summary

The detrimental effects of extracellular histones under pathological conditions have lately attracted considerable attention. However, little is known about their functions as damage-associated molecular pattern molecules. Our study shows for the first time that extracellular histones trigger the induction of chemotactic chemokines from monocytes. As this interaction is dependent on a pattern recognition receptor, namely toll-like receptor 4, our data indeed point to an important role of extracellular histones in danger signaling. Notably, CXCL9 and CXCL10 are chemoattractants, and the recruitment of immune cells to the site of histone injection in a subcutaneous mouse model supports the concept that low levels of extracellular histones constitute a part of the host response.

## Introduction

The rapid response to infections or tissue injury is one of the main features of the innate immune system. To this end pattern recognition receptors (PRRs) play an important role in these processes. PRRs can be divided into different groups or classes such as toll-like receptors (TLRs), C-type lectin receptors, scavenger receptors, and complement receptors [1–3]. Many PRRs are exposed on the host immune cells and they are evolutionary developed to target conserved non-self patterns (pathogen-associated molecular patterns, PAMPs). However, apart from sensing microbial signatures, PRRs can also bind and respond to host derived danger signals [1–3], also referred to as damage-associated molecular patterns (DAMPs). Upon binding to PRRs, DAMPs can trigger several signaling pathways which in turn can lead to a production of pro-inflammatory mediators, including cytokines, chemokines, and vasoactive peptides [4–6]. DAMPs thereby act as adjuvants that further increase the host response in addition to the initial cause of inflammation. One important group of DAMPs consists of the extracellular histones. Histones are ubiquitous proteins that are mainly involved in organizing DNA into nucleosomes and chromatin. Therefore they normally have only an intracellular function and are not actively released into the extracellular environment. However, during inflammatory and/or necrotic conditions histones can be mobilized from stressed, damaged, or dying cells. Such complications are for instance seen in patients with malignant tumors, trauma associated lung injuries, malaria, or severe infectious diseases [7–10].

Several studies have reported that intravenous injection of high histone doses (10–50 mg/kg) causes severe thrombocytopenia and tissue damage in mice and at even higher concentrations (75 mg/kg) death within minutes [11, 12]. Subsequent *in vitro* and *in vivo* experiments have shown that at such concentrations, extracellular histones can evoke an aggregation of platelets, a formation of thrombi, exposure of phosphatidylserine on erythrocytes, and cell necrosis [11–18]. (For a review [19]). By employing knockout animals (TLR2, TLR4, TLR9, and MyD88), extracellular histones have also been found to induce release of pro-inflammatory cytokines in mice (interleukin-6 (IL-6), IL-8, and tumor necrosis factor-α (TNF-α)). Notably, extracellular histones can be found in complex with DNA which has been reported to enhance their immunostimulatory and immunogenic properties [20, 21]. Though these results clearly point to the involvement of toll-like receptors, a characterization of the interaction between histones and TLRs at protein chemical level has not been described. Further, it has not been reported whether histones are able to induce the release of other mediators (for instance substances with chemotactic activities).

The present study was undertaken to analyze a potential role of extracellular histones as sentinels in innate immunity. We show that histone H4 binds directly to the TLR4/myeloid differentiation factor 2 (MD-2) complex and that extracellular histones specifically target monocytes in human peripheral blood. As a consequence, monocytes release the non-ELR CXC chemokines CXCL9 and CXCL10, respectively. In addition, *in vivo* experiments show that this leads to a recruitment of leukocytes. Together our findings implicate an important role of extracellular histones in evoking the innate immune system by sensing danger and damage signals without causing harmful effects for the host.

## Results

### Extracellular histones induce the release of chemokines CXCL9 and CXCL10 but not CXCL11

**Fig 1:**
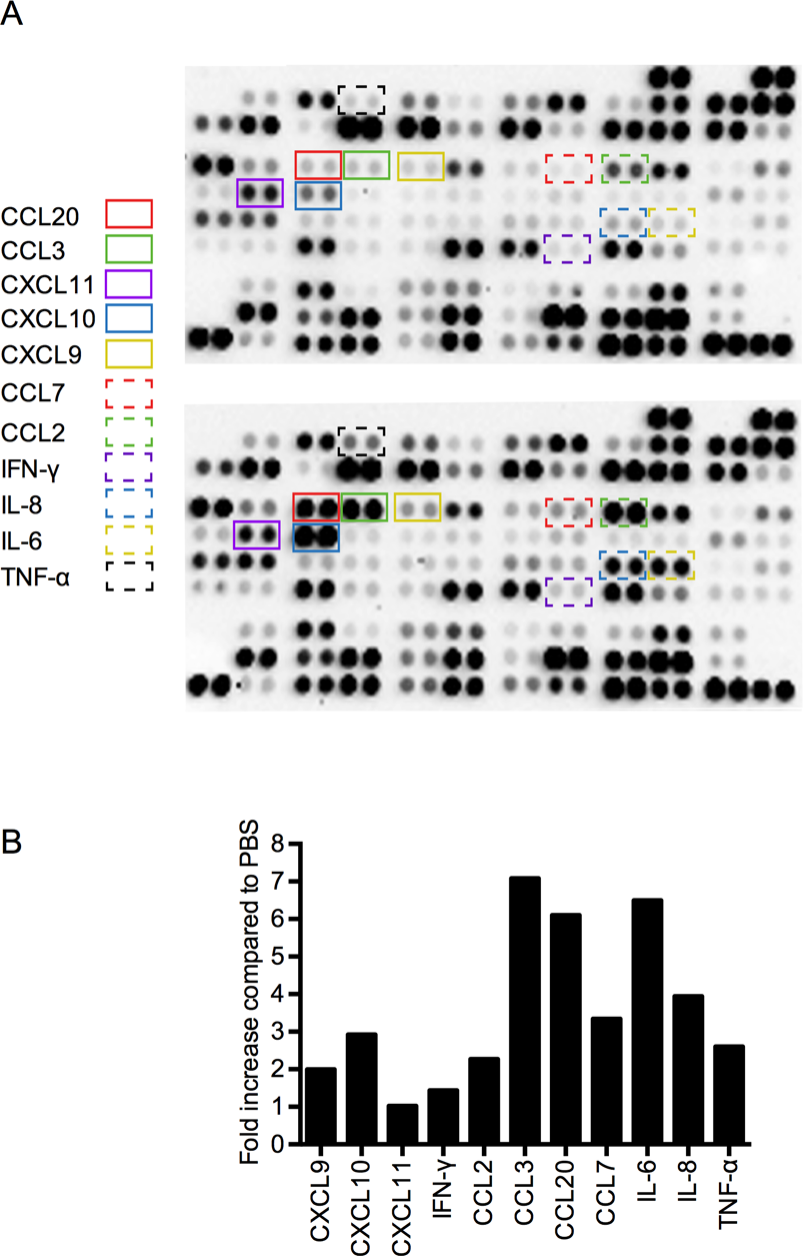
Cytokine imprint of blood stimulated with CTHs. (A) Human heparinized blood was stimulated with CTHs (60 μg/ml) or PBS. After a 12-hour incubation time, samples were centrifuged and the resulting plasma was analyzed using a multi-cytokine membrane array. Mean pixel densities were quantified by an image software and respective cytokine identity is represented as a bar graph. Boxes in different colors and shapes mark cytokines that were found up-regulated (n = 1). (B) Mean pixel densities of cytokine signals were quantified by image software and represented as a bar graph.

In the first series of experiments, we wished to study the role of extracellular histones as potential DAMPs and their ability to induce inflammatory reactions. To this end, calf thymus histones (CTHs) were incubated with human heparinized blood and cytokine levels were determined semi-quantitatively with a multi-cytokine membrane array. A densitometric evaluation of the secreted cytokine pattern revealed that CTH stimulation triggered an increase in the levels of IL-6, IL-8, TNF-α, and IFN-γ when compared to blood incubated with buffer (PBS) alone (Fig 1A). These findings are in line with reports from Xu et al. who studied the release of these mediators in a murine model of inflammation [17]. We also found increased levels of the IFN-γ inducible chemokines, CXCL9 and CXCL10, but not CXCL11, and noted an up-regulation of the chemokines CCL2, CCL3, CCL7, and CCL20, respectively (Fig 1A, boxes).

**Fig 2:**
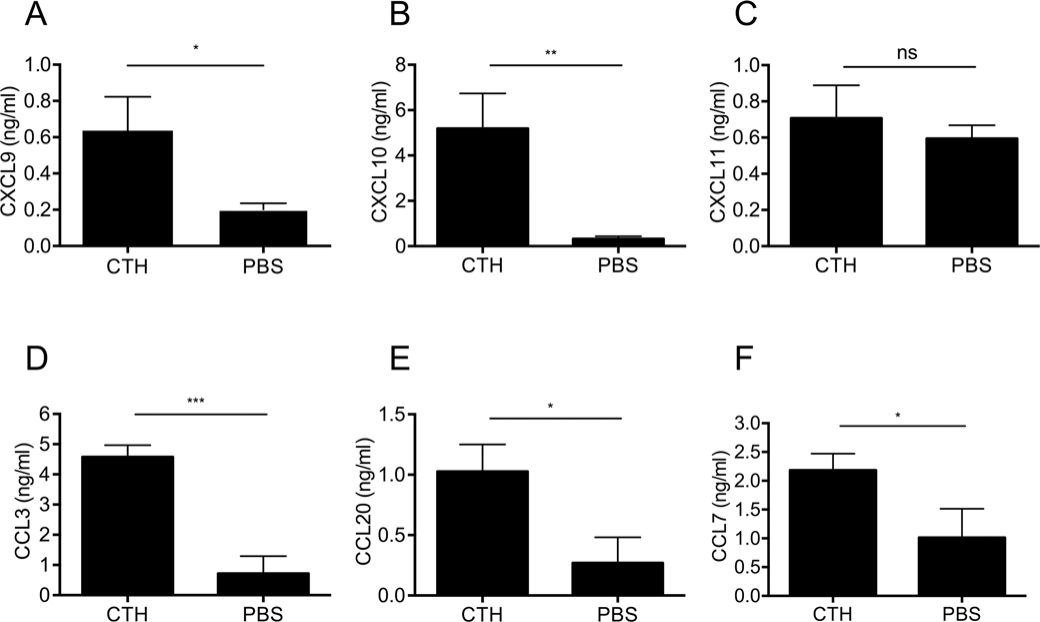
Determination of cytokine levels by ELISA. Human heparinized blood from different healthy donors was incubated with CTHs (60 μg/ml) at 37°C. After a 12-hour incubation the content of CXCL9 (n = 7) (A), CXCL10 (n = 9) (B), CXCL11 (n = 6) (C), CCL3 (n = 4) (D), CCL20 (n = 4) (E), and CCL7 (n = 4) (F) was measured by ELISA.

CXCL9, CXCL10, and CXCL11 belong to the family of non-ELR CXC chemokines that exert their chemotactic activities by binding to CXCR3, a G protein-coupled receptor expressed on monocytes, macrophages, neutrophils, eosinophils, activated T-lymphocytes and NK-cells [22–26]. As the induction of these chemokines by extracellular histones has not been described, the focus was put on these three proteins and in particular on CXCL10 throughout this study. Thus, we investigated the production of the three chemokines over time. Blood from healthy volunteers was treated with CTHs for 12h and the chemokine response was recorded. Plasma levels of CXCL9 and CXCL10 increased significantly (Fig 2A and 2B) while no protein elevation for CXCL11 was observed (Fig 2C). We also found elevated levels of the other three chemokines, CCL3, CCL20, and CCL7 in these samples, suggesting that extracellular histones induce a broad immune response (Fig 2D–2F).

Further analysis of the non-ELR CXC chemokines revealed that the CXCL10 concentration reached a plateau after an incubation time of 10h, while the CXCL9 levels continued to increase even up to 12h. This was in contrast to the levels of CXCL11 which remained constant low over time (S1A Fig). The release of CXCL10 in blood was dose-dependent (S1B Fig) and did not cause cell damage as measured by the release of LDH (S1C Fig). However, at histone concentrations exceeding 50 μg/ml LDH release from PBMCs was recorded (S1D Fig). Together the data show that extracellular histones can trigger the mobilization of chemokines in human blood.

### Histone-induced CXCL10 production is dependent on TLR4 and IFN-γ

**Fig 3:**
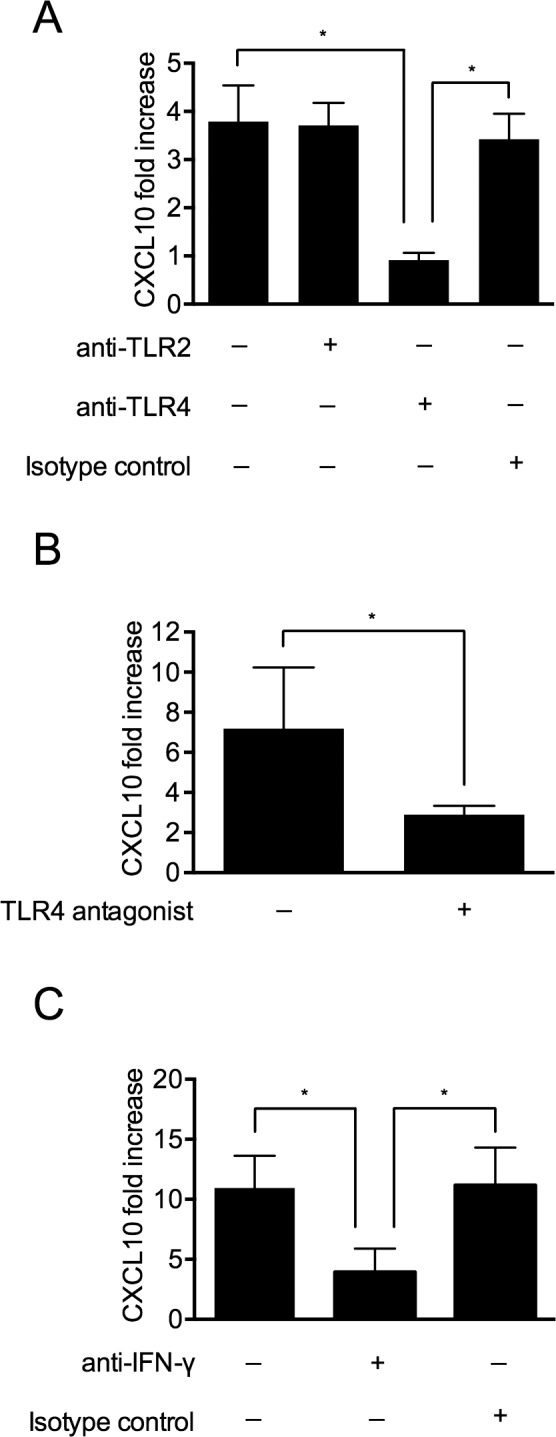
Histone-induced CXCL10 production is dependent on TLR4 and IFN-γ. (A) CTHs (60 μg/ml) were added to heparinized blood in presence of antibodies against human TLR2 (25 μg/ml), human TLR4 (25 μg/ml) or isotype control (25 μg/ml). After a 12-hour incubation at 37°C, the release of CXCL10 was measured. Blood stimulated with CTHs in the absence of an antibody served as control. (B) CTHs (60 μg/ml) were mixed with heparinized blood in the presence or absence of the TLR4 antagonist CLI-095 (1.25 μg/ml). After a 12-hour incubation at 37°C, the release of CXCL10 was measured. Blood stimulated with CTHs in the absence of antagonist served as control. (C) CTHs (60 μg/ml) were incubated with heparinized blood in presence of antibodies against human IFN-γ (25 μg/ml) or an isotype control (25 μg/ml). After a 12-hour incubation at 37°C, the release of CXCL10 was measured. Blood stimulated with CTHs in the absence of an antibody served as control.

Cytokine (IL-6, IL-8, and TNF-α) induction evoked by stimulation with extracellular histones has been reported to involve a preceding activation of TLRs [17]. We therefore sought to investigate if the histone-dependent release of CXCL10 was mediated via TLR signaling. To address this, we used inhibitory antibodies against human TLR2, TLR4, and an isotype antibody as control. The inhibitory activity of the antibodies was first verified by their ability to block the release of interleukin-6 from blood treated with Pam3CSK3 (TLR2 agonist) or LPS (TLR4 agonist), respectively (S2 Fig). In the next series of experiments, CTHs in combination with the anti-TLR2, anti-TLR4, or isotype control antibodies were incubated with heparinized blood for 12h and the release of CXCL10 into plasma was measured. Fig 3A shows that anti-TLR4 significantly inhibited CXCL10 production, whereas anti-TLR2 and the isotype control had no effect. To further confirm that histones induce CXCL10 production through TLR4, the TLR4 antagonist CLI-095 was employed and the CTH-induced CXCL10 production was measured. Also these experiments revealed that the CTH-induced chemokine production is TLR4 dependent (Fig 3B).

The mobilization of CXCL10 is regulated by interferons [27] and incubation of whole blood with extracellular histones can induce the release of IFN-γ (Fig 1). We therefore tested the role of IFN-γ in our experimental model. To this end, human blood was incubated with CTHs in the absence or presence of an inhibitory monoclonal antibody against human IFN-γ or an isotype control. As seen in Fig 3C, CXCL10 production was down-regulated in the presence of the IFN-γ antibody, but not when the control antibody was applied. Together our data suggest that the release of CXCL10 is induced by a chain of events involving an activation of TLR4 that subsequently leads to activation of the IFN-γ receptor, resulting in the expression of chemokines.

### Histone H4 binds to TLR4/MD-2 on monocytes

**Fig 4:**
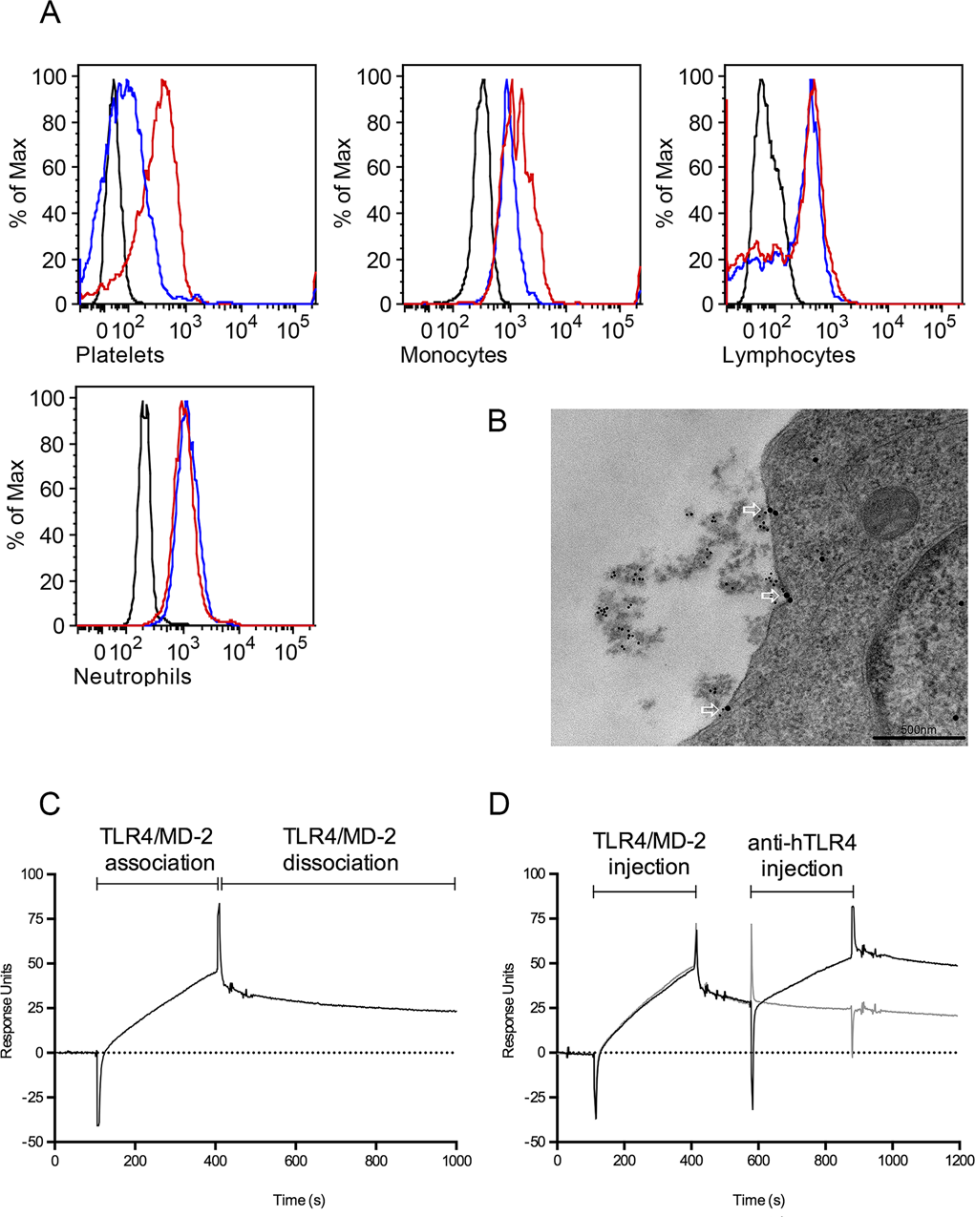
Histone H4 interacts with TLR4/MD-2 on monocytes. (A) Human blood was incubated with histone H4 (red curves) or PBS (blue curves) for 30 min at 37°C. Cells were fixed and stained with a primary antibody against histone H4 followed by a FITC-conjugated secondary antibody. Cells were subjected to flow cytometry analysis and gated for platelets (*upper left*), monocytes (*upper middle*), lymphocytes (*upper right*) and neutrophils (*lower left*). Histone stimulated unstained cells are shown in black curves. (B) THP-1 cells were incubated with histone H4 for 30 min at 37°C. Cells were then fixed and prepared for transmission electron microscopy. Samples were first labeled with rabbit anti-histone H4 and rat anti-TLR4 followed by gold-conjugated goat anti-rabbit (5 nm) and goat anti-rat (15 nm). Arrows indicate co-localization of histone H4 (5 nm gold particles) and TLR4 (15 nm gold particles). Scale bar: 500 nm. (C) Histone H4 was immobilized on a CM5 sensor chip and TLR4/MD-2 complex was applied in a flow. (D) The TLR4/MD-2 complex was injected and after a 580 s dissociation time, an antibody against TLR4 (black) or an isotype control antibody (grey) was applied.

Having characterized the mechanisms that lead to an up-regulation of histone-induced CXCL10 in human blood, we next wished to identify the cell populations that are involved in these processes. We decided to focus on histone H4, since we and others have shown that histone H4 interacts with many cell types including endothelial cell, platelets, and erythrocytes [12, 18]. To study the binding to human blood cells, histone H4 was added to blood and after fixation, cell-bound protein was detected with an antibody against histone H4 followed by a FITC-conjugated secondary antibody. Antibody binding was then recorded by flow cytometry analysis. Histone H4 interacts with monocytes and platelets, while no binding to neutrophils or the lymphocyte population was observed (Fig 4A). To investigate whether the interaction of histones and TLR4 takes place at the plasma membrane of monocytes, histone H4 was incubated with THP-1 monocytes. Cells were double immunostained with gold-labeled antibodies against histone H4 and human TLR4 respectively, and analyzed by transmission electron microscopy. TLR4 and exogenous histone H4 were found in close proximity (Fig 4B), suggesting that this interaction is responsible for the activation of monocytes. Additional surface plasmon resonance experiments were conducted to confirm the binding of TLR4 to histone H4. A sensorchip was immobilized with histone H4 and a flow of TLR4 in complex with its co-factor MD-2 was applied (Fig 4C). An antibody against human TLR4 was then used to confirm that the receptor and not only MD-2 had bound to histone H4 (Fig 4D). Together our results show that histone H4 binds TLR4/MD-2 on monocytes.

### Histones induce CXCL10 production in monocytes

**Fig 5:**
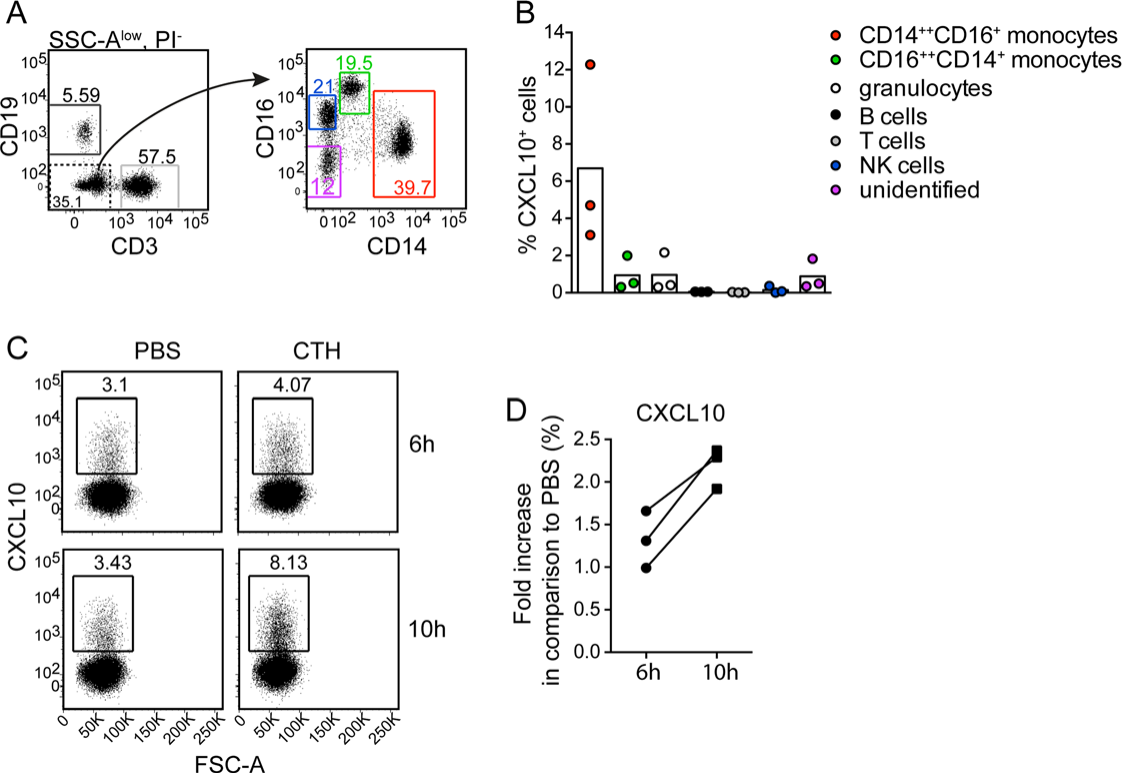
Histones induce CXCL10 production by CD14^++^ CD16^+^ monocytes. Human PBMCs was stimulated with CTHs (10 μg/ml), or PBS for 6h or 10h respectively, with Brefeldin A being present during the last 4h of stimulation. Cells were collected and analyzed for intracellular CXCL10. (A) Gating strategy used to identify indicated peripheral blood cellular subsets after exclusion of granulocytes based on high SSC signal (SSC-A^high^). Cells with a CD16^+^CD14^-^ phenotype were confirmed to be NK cells based on CD56 expression. (B) Percentage CXCL10^+^ cells within indicated subsets after 10h of CTH-stimulation. Background (PBS control) is subtracted. Data from three separate experiments are shown. (C and D) CXCL10 expression by CD14^++^ CD16^+^ monocytes after 6h and 10h stimulation with CTHs or PBS. Representative FACS plots (C) and pooled results, from three separate experiments showing CXCL10 expression in CTH-stimulated cultures relative to PBS control cultures for indicated time-points (D).

To exclude involvement of other cell types than monocytes for CXCL10 production, human PBMCs were separated from neutrophils using Polymorphprep. Isolated PBMCs were then stimulated with CTHs for 6 and 10h and analyzed for intracellular CXCL10. A gating strategy for cellular subsets was defined (Fig 5A), where CXCL10 production was found in CD14^++^CD16^+^ monocytes, while little or no CXCL10 was produced in response to CTHs in the other cell populations (Fig 5B). The production of CXCL10 started to appear after 6h and was clearly induced at 10h (Fig 5C and 5D) which is consistent with other results (S1A Fig). Beyond this, no CXCL10 was produced in neutrophils (S1E Fig). Thus, these findings clearly demonstrate that monocytes are crucial for histone-induced CXCL10 production.

### Mapping of the cell-binding site in histone H4

**Fig 6:**
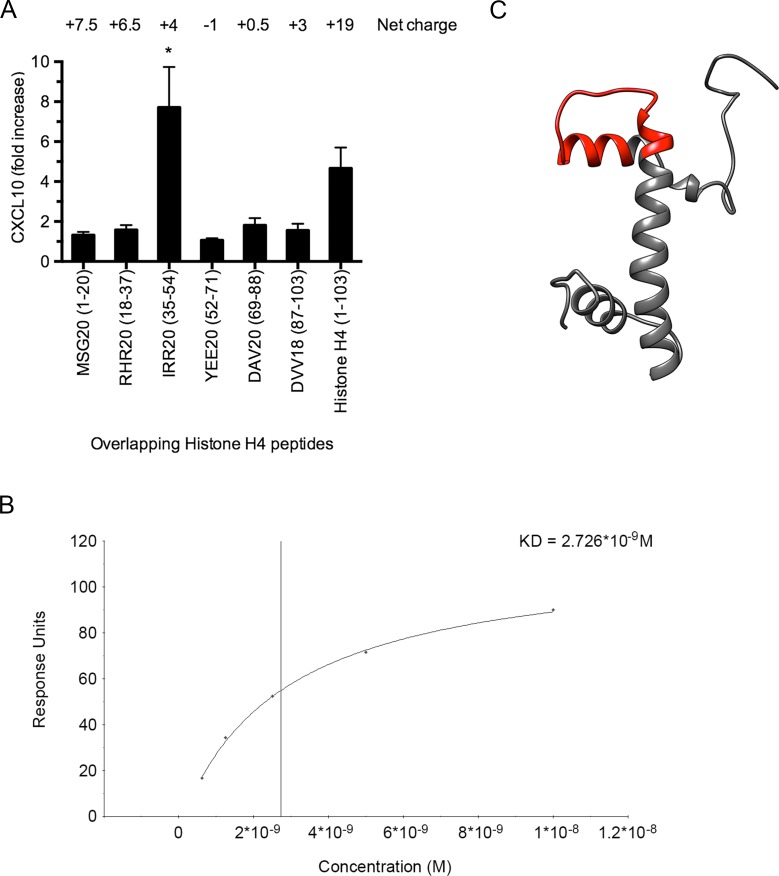
Histone H4 binds to TLR4/MD-2 on THP-1 cells. (A) Overlapping peptides from histone H4 (100 μM) were incubated with heparinized blood for 12h at 37°C and CXCL10 release was measured by ELISA. (B) IRR20 was immobilized on a CM5 sensorchip and serial dilutions (200 nM – 12.5 nM) of the TLR4/MD-2 complex were applied in a flow. Steady-state affinity analysis was used to calculate the KD value (BIAevaluation software, GE Healthcare, Uppsala, Sweden). (C) The predicted structure of histone H4 was modeled using the UCSF Chimera software (http://www.cgl.ucsf.edu/chimera) with a hairpin loop corresponding to the TLR4-binding 20-mer peptide IRR20 marked in red.

Next, we wished to map the cell-binding site in histone H4. Therefore, a panel of overlapping histone H4-derived peptides was incubated with heparinized blood and the induction of CXCL10 was monitored. These results revealed that the cell-binding site is located at amino acids 35–54 (IRR20) towards the amino terminal portion of the protein (Fig 6A). To measure the affinity of the interaction between peptide IRR20 and TLR4/MD-2, surface plasmon resonance experiments were performed. IRR20 was immobilized to a CM5 sensorchip and TLR4/MD-2 was applied in a flow over the surface. Steady-state affinity analysis showed that IRR20 binds to TLR4/MD-2 with high affinity (KD = 2.726 nM) (Fig 6B). Additional modeling suggests that the binding segment forms a hairpin loop surrounded by α-helices (Fig 6C).

### CXCL9 and CXCL10 are elevated in the circulation of mice injected with CTHs

**Fig 7:**
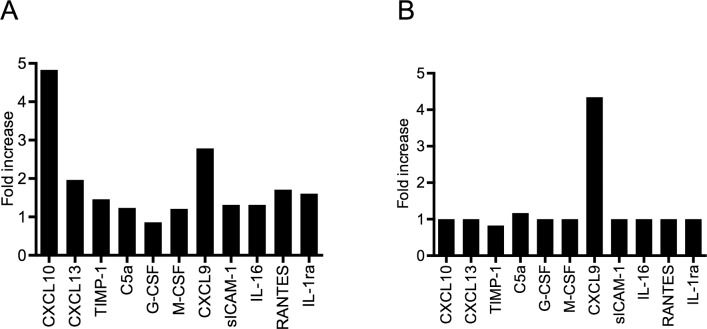
Intravenous injection of CTHs triggers the systemic release of CLXC9 and CXCL10. CTHs (25 mg/kg body weight) or PBS was injected intravenously into Balb/c mice (5 mice per group). After 4h (A) and 24h (B), mice were euthanized and blood samples were recovered by cardiac puncture followed centrifugation. The resulting plasma samples from each group were pooled and analyzed using a mouse multi-cytokine array (n = 1). Mean pixel densities were quantified by Image Lab 4.1 (Bio-Rad Laboratories, Berkeley, CA, USA).

To elucidate the patho-physiological relevance of our findings, an *in vivo* model was used. CTHs were intravenously injected into 10 female Balb/c mice and blood samples were recovered from the animals by cardiac puncture at two time points (4h and 24h respectively). The blood was then centrifuged and samples from 5 animals in each group were pooled. The release of various inflammatory mediators into the plasma was measured in a multi-cytokine array. As shown in Fig 7A, CXCL10 levels increased up to five fold and CXCL9 levels up to 3 fold, respectively, when plasma samples were analyzed after a 4-hour incubation with CTHs. While the CXCL10 concentration decreased to background levels after the 24-hour treatment, CXCL9 increased further and reached levels that were about five times higher than in plasma samples from non-treated mice (Fig 7B). These findings suggest that CXCL9 and CXCL10 production occurs *in vivo* as a response to intravenous injections of CTHs.

### Histones induce recruitment of leukocytes *in vivo*

**Fig 8:**
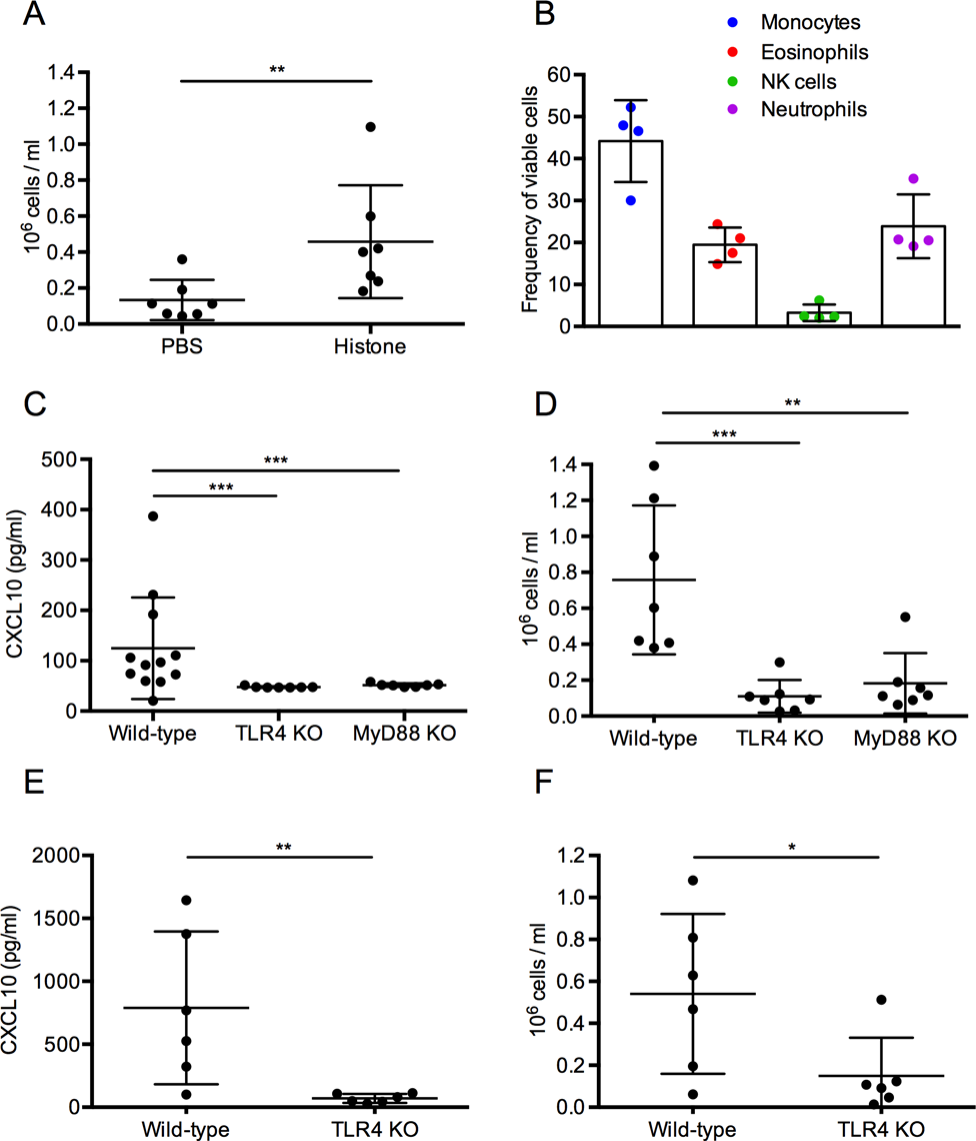
Subcutaneous injection of CTHs induces leukocyte recruitment and local production of CXCL10. (A) Balb/c mice (CTHs (n = 7) and PBS (n = 7)) were challenged with 100 μl of a 10 μg/ml CTHs solution and the number of leukocytes that infiltrated the air pouch were measured. (B) Identification of cellular subsets recruited into the mice air pouch was done as described in Material and Methods. Monocytes (CD11b^+^Ly6C^+^CD64^+^), neutrophils (CD11b^+^Ly6G^+^), eosinophils (CD11b^-^CD64^-^Ly6C^int^SSC-A^high^) and NK cells (CD11b^-^Ly6G^-^NK1.1^+^) were identified after gating on PI^-^ CD3^-^ B220^-^ events. (C) Mice received an injection with 100 μl of a 10 μg/ml CTH solution and the air pouch supernatants from C57BL/6 wild-type (n = 12), TLR4 KO (n = 7), and MyD88 KO (n = 7) mice were used to measure the local levels of CXCL10. (D) Mice received an injection with 100 μl of a 10 μg/ml CTH solution and the number of leukocytes that infiltrated the air pouch, are depicted for wild-type (n = 7), TLR4 KO (n = 7), and MyD88 KO (n = 7) and mice. (E) Mice received an injection with 100 μl of a 100 μM IRR20 solution and the air pouch supernatants from wild-type (n = 6) and TLR4 knockout (n = 6) mice were used to measure the concentration of CXCL10. (F) Mice received an injection with 100 μl of a 100 μM IRR20 solution and the number of leukocytes that infiltrated the air pouch, was determined in wild-type (n = 6) and TLR4 knockout (n = 6) mice.

CXCL9 and CXCL10 have been reported to act as chemoattractants for cells expressing the CXCR3 receptor, such as monocytes, macrophages, neutrophils, eosinophils, activated T-lymphocytes and NK-cells [22–26]. To test whether the local injection of histones into Balb/c mice leads to a recruitment of such immune cells, we employed a CTH air pouch model (see Material and Methods). Mice receiving a PBS injection instead of CTHs served as control. Sixteen hours after injection, fluids from the air pouch were collected and analyzed in the presence of counting beads by flow cytometry. Fig 8A depicts the increased cellular influx when animals were treated with CTHs. Further flow cytometry analysis revealed that different populations of leukocytes recruited into the air pouch are monocytes, eosinophils, NK-cells, and neutrophils, respectively (Fig 8B). As these cells have been reported to express the CXCR3 receptor [22–26] we next tested whether the recruitment of these cells correlates with the CXCL10 levels in the air pouch. For this purpose C57BL/6 wild-type mice received a subcutaneous injection of CTHs and the CXCL10 levels in the air pouch were measured after 16h of incubation. TLR4 and MyD88 knockout animals were used to confirm that the histone-induced release of CXCL10 is dependent on TLR4 receptor signaling. As shown in Fig 8C, increased levels of the chemokine were recovered from the air pouch of wild-type, but not of TLR4 and MyD88 knockout mice. Consequently, also the influx of leukocytes into the air pouch was significantly reduced in the knockout animals when compared to wild-type animals (Fig 8D). To exclude a LPS contamination in the CTH preparation, the synthetic histone H4-derived peptide IRR20 was injected in wild-type and TLR4 knockout mice. As seen for CTHs, IRR20 was able to trigger the induction of CXCL10 (Fig 8E) and recruitment of leukocytes (Fig 8F) in wild-type but not TLR4 knockout mice. Together these experiments show that subcutaneous injection of extracellular histones leads to the local release of CXCL10 and recruitment of leukocytes to the side of injection.

### CXCL10 production in *S*. *pyogenes* infection is dependent on endogenous extracellular histones

**Fig 9:**
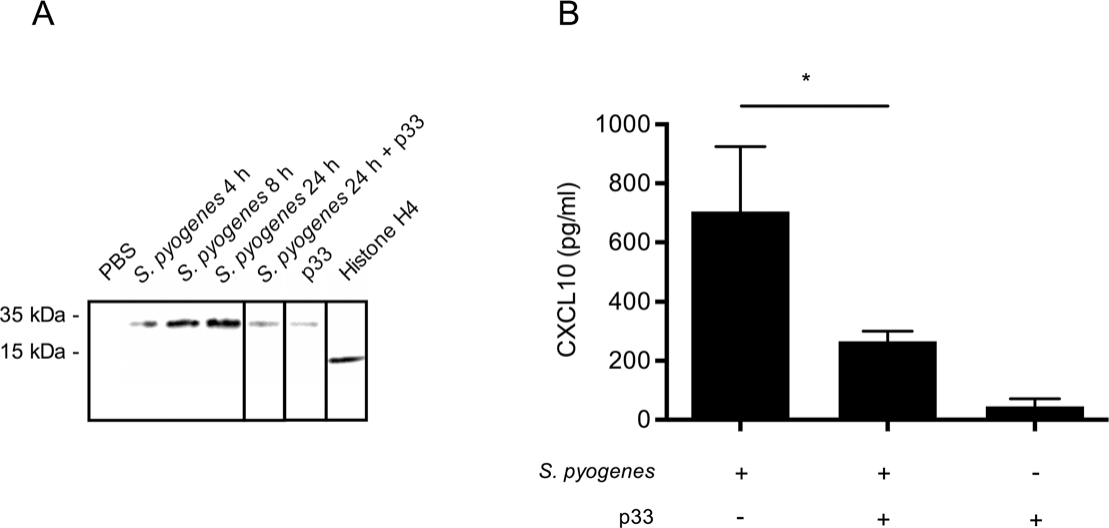
Histone H4 and CXCL10 expression after *S*. *pyogenes* infection. (A) Histone H4 was immunostained by Western blot analysis in the supernatants from air pouch of C57BL/6 wild-type mice inoculated with *S*. *pyogenes* (AP1 strain) for 4h, 8h, and 24h. p33 treatment was administrated 8h after infection. Note that the shift in the molecular weight of histone H4 recovered from the air pouch compared to recombinant histone H4 is due to histone acetylation and/or methylation [28]. (B) CXCL10 was measured by ELISA in the supernatant from air pouch of C57BL/6 wild-type mice inoculated with *S*. *pyogenes* (AP1 strain, n = 3), infected mice treated with p33 8h after infection (n = 3), and mice injected with p33 only (n = 3).

To confirm that extracellular histones are important for chemokine production during *S*. *pyogenes* infection, we inoculated C57BL/6 wild-type mice with *S*. *pyogenes* by subcutaneous injection. Accumulation of extracellular histone H4 in the air pouch was detected by Western blot analysis and Fig 9A depicts that increasing concentrations were measured 4h, 8h, and 24h after infection, respectively. We recently reported that p33, also known as gC1q receptor is a specific inhibitor of extracellular histones [12]. To test whether p33 can interfere with the chemotactic activity of the extracellular histones, the protein was injected into the air pouch 8h after infection. Subsequent Western blot analysis revealed that this treatment let to a reduction of histones in the air pouch which was in a similar low range as seen in animals that were treated with p33 for 24h in the absence of bacteria (Fig 9A). No histones were detected in air pouches of mice that were injected with PBS only. Purified recombinantly produced histone H4 was used to show the specificity of the antibody.

Having shown that extracellular histones are generated during infection, we next investigated whether this leads to an induction of CXCL10 in the air pouch. Fig 9B shows that elevated concentrations of the chemokine are detected in mice infected for 24h with *S*. *pyogenes*. However, when the mice were treated with p33, the levels of CXCL10 were significantly decreased and only minor CXCL10 concentrations were found in non-infected animals that received a p33 injection (Fig 9B). Together these findings suggest that endogenous extracellular histones were responsible for the CXCL10 production in the infected mice.

### Histone H4 and CXCL10 are mobilized in patients under infectious disease conditions

**Fig 10:**
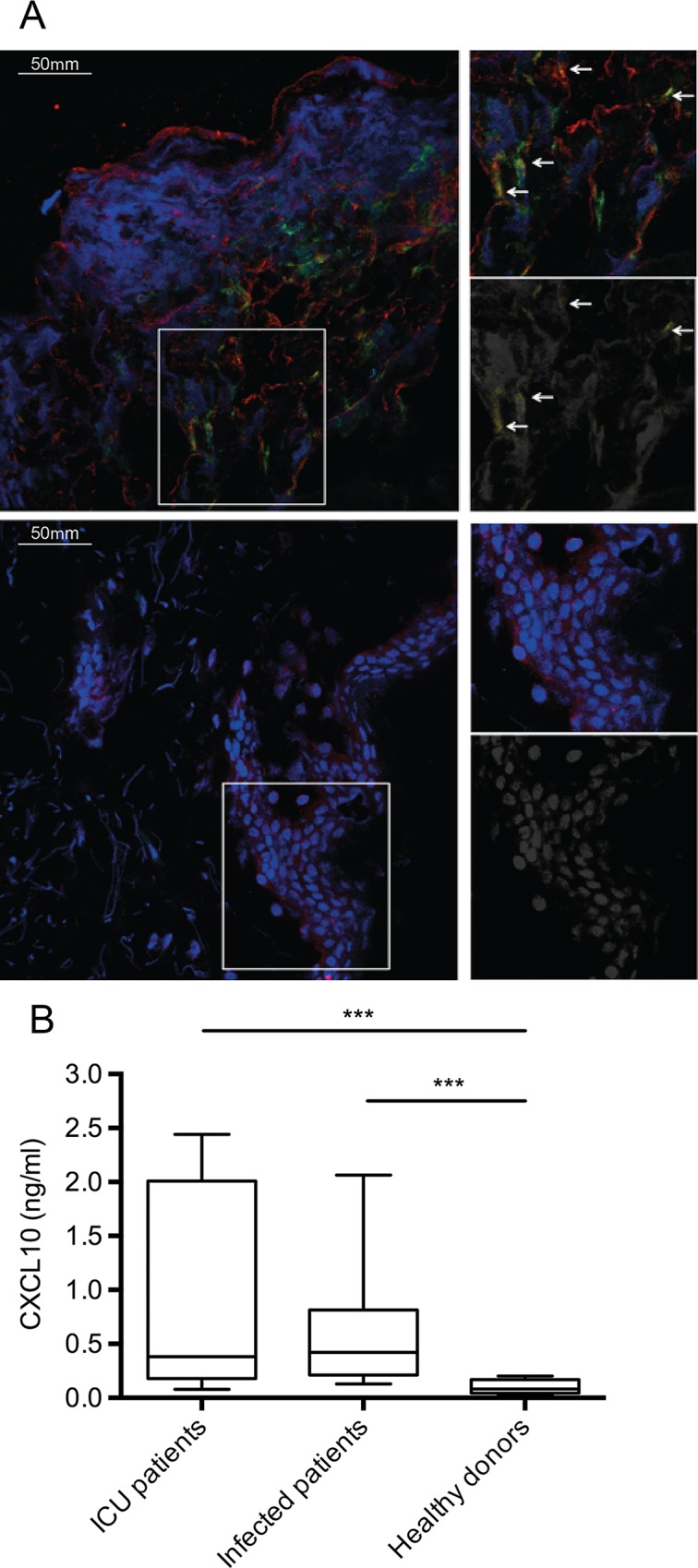
Detection of extracellular histone H4 and CXCL10 in patient material. (A) Immunostaining of snap-frozen tissue biopsies from a patient with *S*. *pyogenes* deep tissue infection (*upper level*) and a healthy donor (*lower level*) are depicted. Histone H4 staining is shown in red, CXCL10 in green, and DNA (DAPI) in blue. The boxed areas provide a larger magnification (*right side*). Co-localization of histone H4 and CXCL10 is shown in yellow and indicated by arrows. The image shows staining of one representative patient biopsy out of three analyzed. (B) CXCL10 was measured in the blood plasma from patients admitted at the ICU suffering from septic shock or non-septic critical illness (n = 15), patients with milder infections (upper respiratory tract viral infections or gastroenteritis) (n = 15) and healthy individuals (n = 10).

Our previous results show that histone H4 is released into the tissue during severe deep tissue infections such as necrotizing fasciitis and cellulitis [12]. Thus, we wished to study whether the release of extracellular histones co-localizes with the induction of CXCL10 in patients suffering from *S*. *pyogenes* tissue infections. Immunofluorescence microscopy was used to analyze tissue biopsies from patients with severe deep tissue infections, i.e. severe cellulitis or necrotizing fasciitis, evoked by *S*. *pyogenes*. Fig 10A (*upper panel*) illustrates that the infection caused necrotic tissue leading to severe cell damage. Further immunostaining analysis revealed the mobilization of extracellular histone H4 and CXCL10 and at some locations co-localization of the two factors was noted (Fig 10A, arrows, yellow). In the healthy tissue, only intact cells and consequently also only intracellular DNA was seen. We were therefore not able to co-localize histone H4 or CXCL10 in these tissue samples (Fig 10A, lower panel).

To investigate if CXCL10 is systemically up-regulated during infection, the levels of the chemokine were measured in plasma samples from patients admitted at the intensive care unit (ICU) suffering from septic shock or non-septic critical illness. For comparison samples from patients with milder infections (upper respiratory tract viral infections and gastroenteritis) or from healthy individuals were used. The highest levels of CXCL10 were found in plasma samples from ICU patients (Fig 10B). Though CXCL10 concentrations in plasma samples from patients suffering from mild infection were lower than in ICU patients, their levels were still significantly increased compared to those measured in samples from healthy donors (Fig 10B). Taken together, the patient data may support our *in vitro* and *in vivo* findings that the release of histones induces the induction of CXCL10 and that patients with severe disease conditions have a systemic increase of the chemokine.

## Discussion

The role of extracellular histones in the pathology of severe diseases such as sepsis has lately attracted considerable attention [10, 11, 15–17, 19, 29, 30]. Extracellular histones are released into the surrounding tissue or circulation from NETotic/necrotic cells and at high concentrations they can trigger toxic responses and evoke systemic inflammatory reactions. This in turn may lead to life-threatening complications including disturbed hemostatic functions and acute organ injury [19]. Animal studies have shown that mice receiving sub-lethal doses of histones develop symptoms that are also often seen in patients suffering from severe infectious diseases [11, 15, 17, 19]. These findings point to an important role for extracellular histones in disease progression. However, little is known whether the release of lower histone levels is less devastating and perhaps even could contribute to host reactions that are involved in immune defense or wound healing.

In the present study we sought to address this issue by studying if extracellular histones can act as danger signals by triggering local host responses. Our data indeed supported this notion, as histones induced the production of the IFN-γ inducible chemokines, CXCL9 and CXCL10, respectively. Both chemokines are important chemoattractants [23, 31] and our results show that the subcutaneous injection of extracellular histones evokes an influx of inflammatory cells to the challenged sites. Further characterization revealed that histones specifically target monocytes by approaching a pattern recognition receptor, namely TLR4 and *in vivo* experiment with TLR4 and MyD88 knockout mice confirmed the involvement of the receptor. Additional examination of tissue biopsies from patients with tissue infections depicted co-localization of extracellular histones and CXCL10, suggesting that the chemokine is released at the site of infection under disease conditions. When analyzing plasma samples from patients suffering from infectious diseases, we found that the increase of CXCL10 in these samples correlated well with the severity state of the disease. However, unlike described for other inflammatory mediators such as IL-6 and TNF-α [17] CXCL10 concentrations did not reach pathological levels within the microgram range (cytokine storm). We can therefore conclude that extracellular histones are able to cause local and systemic release of IFN-γ inducible chemokines, however, under systemic conditions it seems that these chemokines do not contribute as much to disease progression as other cytokines.

It is noteworthy to mention that analyzing the cytokine release pattern of murine blood samples revealed that CXCL9 and CXCL10 are the most abundant inflammatory mediators detected when mice were injected with CTHs. Notably, both chemokines have in addition to their chemotactic activities other features. For instance, CXCL9 and CXCL10 have been reported to carry antimicrobial activity [32, 33] and are pro-apoptotic [34]. The two effects have in common that they help prevent an induction of systemic inflammatory complications. This further strengthens our concept that the two chemokines are released in response to a danger signal and are part of a local immune response. Taken together we here provide for the first time evidence that histones can act as DAMPs/alarmins through inducing leukocyte migration and trigger the release of the IFN-γ inducible chemokines, CXCL9 and CXCL10. These findings demonstrate a dual role of extracellular histones in local and systemic disease conditions. It will be important to understand the details of this dual role in future development of therapies targeting extracellular histones.

## Materials and Methods

### Ethics statement

The study was approved by the Institutional review board (IRB) at the Lund University Hospital (Protocol #790/2005). Plasma samples and tissue biopsies (Lund University Hospital, Sweden and University of Toronto, Canada) were taken with a written informed consent, and the study included patients above 18 years of age. Animal use protocols (#M108-10, #M326-12 and #M327-12) were approved by the local Institutional Animal Care and Use Committee (IACUC) of Malmö/Lund, Sweden. All animals were handled according to the Swedish Animal Welfare Act (SFS 1988:534). The Institutional Review Boards (IRBs) of the University of Toronto (Toronto, ON) and Karolinska University Hospital (Stockholm, Sweden) approved the studies involving humans, and written informed consent was obtained from the patients and the volunteers.

### Animals

Female and male Balb/c, C57BL/6 wild-type and knockout mice were purchased from Charles River (Sulzfeld, Germany) and were at least 8 weeks old at the initiation of the experiments.

### Proteins and antibodies

Bovine calf thymus histones (CTHs) were purchased from Roche (Basel, Switzerland). Overlapping Histone H4-derived peptides were synthesized at Biopeptide Co. (San Diego, CA, USA). CTHs have been used by researchers for both *in vivo* and *in vitro* studies of the function of extracellular histones [11, 12, 15–18, 20]. LPS-free CTHs and LPS-free histone H4-derived peptides were dissolved in LPS-free water. Recombinant histone H1, H2A, H2B, H3.1 and H4 were purchased from New England Biolabs (Ipswich, MA, USA). Recombinant human TLR4/MD-2 complexes were obtained from R&D Systems (Minneapolis, MN, USA) Peroxidase-conjugated goat anti-rabbit and goat anti-mice immunoglobulin G was from Bio-Rad Laboratories (Berkeley, CA, USA). Antibodies against histone H4 (polyclonal chip-grade anti-histone H4) were from Abcam (Cambridge, UK). Rat polyclonal antibodies to human TLR4, TLR2 and isotype control were purchased from Invivogen (San Diego, CA, USA). Mouse anti-human interferon-γ (clone B27) and isotype control were purchased from BioLegend (San Diego, CA, USA). Pam3CSK was obtained from Invivogen (San Diego, CA, USA) and LPS from *Salmonella enterica* serotype typhimurium from Sigma-Aldrich (St. Louis, MO, USA).

### Patient samples

Plasma samples from 30 patients enrolled at Lund University Hospital, Sweden were analyzed. Fifteen patients treated in the ICU due to septic shock (n = 8) and non-septic critical illness (n = 7) and another 15 patient with milder infections such as upper respiratory tract viral infections (n = 10) and gastroenteritis (n = 5) were enrolled. Blood samples for the analyses were collected at enrollment in 5 ml plastic vacutainer tubes containing 0.5 mL of 0.129 mol/l sodium citrate.

### Blood collection

Peripheral venous blood samples were collected from healthy human donors into 6.0 ml sodium heparin tubes (102 IU) or 2.7 ml 0.109 M buffered sodium citrate (Becton Dickinson, Franklin Lakes, NJ, USA). Blood samples were immediately used for experiments upon collection.

### LPS measurement in histones

The presence of LPS was measured in all histone subclasses, histone H4-peptides and CTHs using the Pierce LAL Chromogenic Endotoxin Quantitation Kit (ThermoFisher Scientific, MA, USA) according to manufacturer’s protocols. Histone H2A, H2B and H3.1 contained significant levels of LPS (S3A Fig), while no LPS was found in histone H4, CTHs or histone H4-derived synthetic peptides (S3B Fig). Therefore, only CTHs, histone H4 and histone H4-derived peptides were used throughout this work.

### Cytokine arrays

Human XL Cytokine Array Kit and Mouse Cytokine Array Panel A were purchased from R&D Systems (Minneapolis, MN, USA) and experiments were performed according to the manufacturer’s protocols. Human heparin blood was stimulated with CTHs (60 μg/ml, 12 h) centrifuged (2000 x g for 10 min) and the resulting plasma was used for the Human XL Cytokine Array Kit. Mice were injected with CTHs (25 mg/kg body weight, 4 h) and euthanized with CO_2_ inhalation followed by cervical dislocation. Blood was drawn by cardiac puncture using a syringe filled with 70 μl of buffered sodium citrate and then transferred into K2 Microtainer MAP tubes pre-coated with 1 mg EDTA (Becton Dickinson, NJ, USA). Blood was centrifuged and the mouse plasma was used for the Mouse Cytokine Array Panel A.

### Surface plasmon resonance

Analyses were performed with a BIAcore X100 instrument (GE Healthcare, Uppsala, Sweden) using Sensor Chip CM5 technology at 25°C in a HBS-EP buffer (10 mM HEPES, 150 mM NaCl, 3 mM EDTA, 0.05% (v/v) Surfactant P20, pH 7.4). Histone H4 or IRR20 was diluted in sodium acetate (10 mM, pH 5.5) and immobilized via amine coupling to flow cell 2. Flow cell 1 was subjected to the coupling reaction but without protein, and was used as a control in each experiment. TLR4/MD-2 was injected over the coated surface at 30 μl/min in running buffer. Regeneration of sensorchip surfaces was obtained by injection of 30 μl 50 mM NaOH.

### Cytokine production in whole blood

CTHs (60 μg/ml), Pam3CSK4 (200 ng/ml) or LPS (100 ng/ml) were incubated with heparinized blood at 37°C on rotation. At different time points, aliquots were removed and centrifuged at 2000 x g for 10 min. All plasma samples were stored at -20°C for further experiments. For inhibition experiments, the TLR4 signaling inhibitor CLI-095 (1.25 μg/ml) or antibodies with blocking activity against human IFN-γ (25 μg/ml), human TLR2 (25 μg/ml), human TLR4 (25 μg/ml) or polyclonal control antibodies (25 μg/ml) were added simultaneously with 60 μg/ml CTHs, Pam3CSK4 or LPS. Histone H4-derived synthetic peptides were incubated with heparinized blood at 37°C on rotation for 12 h. After centrifugation, CXCL10 was measured in the plasma using Human CXCL10/IP-10 Quantikine ELISA kit from R&D Systems (Minneapolis, MN, USA).

### Cytokine production in peripheral blood mononuclear cells (PBMCs)

PBMCs were isolated from heparinized human blood using Polymorphprep (Axis-Shield, Dundee, Scotland) according to manufacturer’s protocols. PBMCs (8x10^5^ cells/ml) were stimulated with different concentrations of CTHs for 6h, 10h or 12h at 37°C, 5% CO_2_.

### Cytokine ELISA

Cytokine ELISA kits for human CXCL10, mouse CXCL10, human CXCL9 and human CXCL11 were purchased from R&D Systems (Minneapolis, MN, USA) and performed according to manufacturer’s instructions. ELISA kits from R&D Systems were not optimized for plasma samples from citrated blood. Therefore heparinized blood was used for these experiments. Cytokine ELISA kits for human CCL3, CCL20, CCL7 were purchased from Sigma-Aldrich (St. Louis, MO, USA).

### Cytotoxicity assay

Histone-induced cell toxicity was measured in heparinized blood or PBMCs using the Tox-7 kit (Sigma Aldrich, St. Louis, MO, USA). Cells were stimulated with CTHs (0–100 μg/ml) for 12h at 37°C, 5% CO_2_. Cells were pelleted and the supernatants were used for measurement of lactate dehydrogenase (LDH) according to the manufacturers’ protocol. Histone-induced LDH release was calculated as percentage of Tox-7 lysis-induced LDH release.

### Histone-white blood cell interactions

Citrated blood (20 μl) was diluted with 40 μl Cell Staining Buffer (BioLegend, San Diego, CA, USA). CTHs (final concentration 200 μg/ml) were added and samples were incubated for 30 min, 37°C, 5% CO_2_. Cells were washed in 900 μl cell staining buffer, centrifuged (500 x g for 15 min, 4°C) and the pellet was resuspended in 200 μl buffer. Histone H4 bound to cells was stained with 2 μg/ml of a primary rabbit anti-human histone H4 (Abcam, Cambridge, UK) for 30 min on ice. Erythrocytes were lysed first with 200 μl Lysis buffer A (10 min, room temperature, dark) followed by 1 ml Lysis buffer B (10 min, room temperature, dark). Supernatant containing lysed erythrocytes were removed and remaining white blood cells were resuspended in 1 ml cold cell staining buffer. A secondary FITC-conjugated goat anti-rabbit antibody (Abcam, Cambridge, UK) was added (2 μg/ml) and samples were incubated for 30 min on ice in dark. Two more wash steps were performed and each sample was analyzed in flow cytometry.

### Intracellular cytokine staining in human PBMCs

Total PBMCs were cultured with or without CTHs (final concentration 10 μg/ml) in RPMI 1640 for 6h or 10h with 1X Brefeldin A (eBioscience, San Diego, CA, USA) added during the last 4 h. Stimulated cells were collected in cold FACS buffer (PBS supplemented with 2% FCS and 2 mM EDTA) and unspecific binding was blocked with 10% mouse serum. Surface stain was done with the following antibodies; anti-CD19 (HIB19), anti-CD16 (3G8) (BioLegend, San Diego, CA, USA), anti-CD4 (RPAT4) (BD Biosciences, Franklin Lakes, NJ, USA), CD3 (UCHT1), anti-CD8 (RPAT8), anti-CD14 (61D3) and anti-CD56 (CMSSB) (eBioscience, San Diego, CA, USA) conjugated to allophycocyanin-Cy7, FITC, Alexa700, eFluor450, PE-Cy7, allophycocyanin or biotin revealed by streptavidin-PECF594 (BD Biosciences, Franklin Lakes, NJ, USA). Dead cells were excluded with Live/Dead fixable aqua dead cell stain kit (Life Technologies). Intracellular staining was done with the FoxP3 Fixation/Permeabilization kit (eBioscience, San Diego, CA, USA) using anti-IP10 (J034D6) conjugated to PE (BioLegend). Data were collected using a LSRII (BD Biosciences, Franklin Lakes, NJ, USA) and analyzed with the FlowJo software (Tree star, Ashland, OR, USA).

### *In vivo* cell migration

CTHs (100 μl, 10 μg/ml,), IRR20 (100 μM) or PBS were injected subcutaneously into an air pouch of male and female wild-type (C57BL/6), TLR4 knockout (C57BL/6), MyD88 knockout (C57BL/6), or wild-type Balb/c mice. After 16h the air pouch was rinsed with 1 ml PBS and the rinsing solution was fixed in 2% paraform aldehyde for 30 min on ice. CountBright Counting Beads (ThermoFisher Scientific, MA, USA) were diluted 1:10 into the fixed cells and the samples were counted with a FACSVerse Flow Cytometer (Becton Dickinson, NJ, USA). Cells and counting beads were gated with side scatter and forward scatter and analyzed with FlowJo 9.3.1 Software. Identification of cellular subsets was done by flow cytometry in conjunction with mAb against CD64 (X54-5/7.1), CD3 (145-2C11) (BD Biosciences, Franklin Lakes, NJ, USA), NK1.1 (PK136), Ly6G (1A8), Ly6C (HK1.4), CD11c (N418), CD11b (M1/70) (BioLegend), B220 (RA3-6B2) and CD4 (GK1.5) (eBioscience, San Diego, CA, USA). Unspecific binding was blocked using anti-FcR mAb (2.462) and dead cells were excluded with propidium iodide (PI). Data were collected using a LSRII (BD Biosciences, Franklin Lakes, NJ, USA) and analyzed with FlowJo (Tree star). Monocytes (CD11b+Ly6C+CD64+), neutrophils (CD11b^+^Ly6G^+^), eosinophils (CD11b^-^CD64^-^Ly6C^int^SSC-A^high^) and NK cells (CD11b^-^Ly6G^-^NK1.1^+^) were identified after gating on PI^-^ CD3^-^ B220^-^ events.

### Animal model of *S*. *pyogenes* infection

Wild-type C57BL/6 mice were subjected to a subcutaneous infection of *S*. *pyogenes* (AP1 strain) as previously described [35]. Briefly, mice were inoculated with 10^8^ CFU/ml log-phase bacteria, and euthanized at different time points. To remove endogenous histones released after *S*. *pyogenes* infection, mice were treated by administration of p33 (50 μg/ml) 8h after initial infection. After 4 h, 8h or 24 h, the air pouch was rinsed with 1 ml PBS and cells and bacteria were pelleted. Samples were subjected to Western blot analysis in the cell supernatant by pooling samples from mice in the same group. Histone H4 was detected using mouse anti-histone H4 antibody (ab31830, Abcam, Cambridge, UK) and secondary goat anti-mouse HRP (1:1000, Bio-Rad Laboratories Berkeley, CA, USA). CXCL10 levels in the air pouch were measured by ELISA.

### Co-localization Histone H4 and CXCL10

Snap-frozen tissue biopsies collected from the center of infection from three patients with deep tissue infections, i.e. severe cellulitis or necrotizing fasciitis, caused by *S*. *pyogenes* were analyzed (kindly provided by Prof. Donald E Low, Mount Sinai Hospital, Toronto, Canada). Also included were biopsies of healthy skin tissue obtained at plastic surgery at Karolinska University Hospital. The biopsies were cryosectioned to 8 μm, fixed in ice-cold acetone and immunofluorescent stainings of serial sections were conducted as described previously [36]. The following antibodies were used: rabbit polyclonal anti-histone H4 and mouse monoclonal anti-IP10 (clone 6D4) (both from Abcam, Cambridge, UK), Alexa 546 conjugated donkey anti-rabbit IgG and Alexa 488 conjugated donkey anti-mouse IgG (both from Molecular Probes, Eugene, OR, USA). Slides were mounted using DAPI supplemented mounting media (Molecular Probes, Eugene, OR, USA). Single stainings were performed to assure specificity of staining patterns. For image evaluation, a Nikon A1R confocal microscope was used (Nikon Instruments, Amstelveen, the Netherlands).

### Immunoelectron microscopy

Monocyte THP-1 cells were incubated with histone H4 (50 μg/ml) for 30 min at 37°C. For immunohistochemistry and transmission electron microscopy, samples were embedded in Epon resin, sectioned and subjected to antigen retrieval with metaperiodate as recently described in detail [37]. Sections were labeled with rabbit anti-histone H4 and rat anti-TLR4 followed by gold-conjugated goat-anti rabbit (5 nm) and goat-anti rat (10 nm) antibodies. Samples were observed in a Philips/FEI CM 100 transmission electron microscope at the Core Facility for Integrated Microscopy, Panum Institute, University of Copenhagen.

### Statistical analysis

Data were analyzed using GraphPad Prism 6 (GraphPad Software, San Diego, CA, USA). Statistical significance was determined by using the non-parametric Mann-Whitney U test for comparison between two groups. The level of statistical significance was defined as a two-tailed p-value of < 0.05 (*), < 0.005 (**) and < 0.0005 (***).

### ID numbers

CXCL9, Q07325

CXCL10, P02778

CXCL11, O14625

CXCR3, P49682

IFN-γ, P01579

TLR4, O00206

TLR2, O60603

p33, Q07021

Histone H4, P62805

## Supporting Information

S1 Fig Dose dependency of Histone-induced CXCL10 release and LDH release. (A) Human heparinized blood was incubated with CTHs (60 μg/ml) at 37°C. At different time points, the content of CXCL9, CXCL10 and CXCL11 was measured by ELISA. The image shows one representative time curve out of three. (B) Human heparinized blood was incubated with different concentrations of CTHs for 12h at 37°C. CXCL10 levels were measured by ELISA. (C) Human heparinized blood were incubated with different concentrations of CTHs for 12h at 37°C. LDH release was measured in the supernatant. (D) Human PBMCs was incubated with different concentrations of CTHs for 12h at 37°C. LDH release was measured in the plasma supernatant. (E) Human neutrophils were incubated with different concentrations of CTHs for 12h at 37°C. CXCL10 levels were measured by ELISA.
(TIFF)

S2 Fig Dose response curves of CXCL10 induction by different histone subclasses. Pam3CSK4 (200 ng/ml) or LPS (100 ng/ml) was added to heparinized blood in presence of antibodies against human TLR2 (25 μg/ml), human TLR4 (25 μg/ml), or an isotype control (25 μg/ml). After a 12-hour incubation at 37°C, the release of IL-6 was measured. Blood stimulated with CTHs in the absence of an antibody served as control.
(TIFF)

S3 Fig LPS levels in recombinant histones. Traces of LPS were measured in different histone subclasses (A) and histone H4-derived synthetic peptides (B) using Pierce LAL Chromogenic Endotoxin Quantitation Kit.
(TIFF)

S1 Appendix Manuscript raw data. Raw data from the manuscript has been added as an appendix.
(XLSX)
