# Supplementary figures and images for: Extracellular Histones Induce Chemokine Production in Whole Blood Ex Vivo and Leukocyte Recruitment In Vivo
Source: PLoS Pathog. 2015 Dec 8;11(12):e1005319. doi: 10.1371/journal.ppat.1005319 (PMC4672907; doi:10.1371/journal.ppat.1005319)

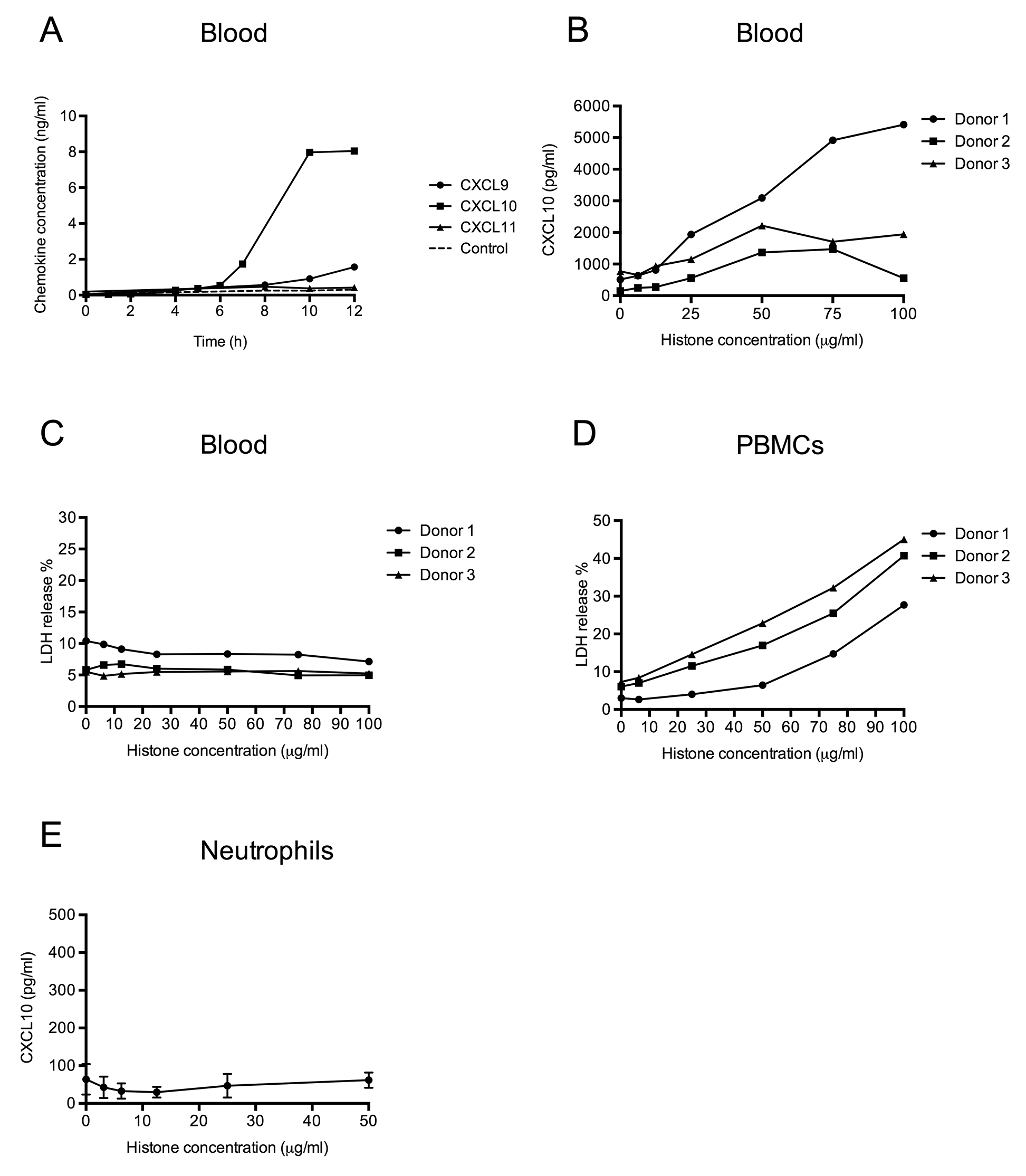

Supplement: S1 Fig — (A) Human heparinized blood was incubated with CTHs (60 μg/ml) at 37°C. At different time points, the content of CXCL9, CXCL10 and CXCL11 was measured by ELISA. The image shows one representative time curve out of three. (B) Human heparinized blood was incubated with different concentrations of CTHs for 12h at 37°C. CXCL10 levels were measured by ELISA. (C) Human heparinized blood were incubated with different concentrations of CTHs for 12h at 37°C. LDH release was measured in the supernatant. (D) Human PBMCs was incubated with different concentrations of CTHs for 12h at 37°C. LDH release was measured in the plasma supernatant. (E) Human neutrophils were incubated with different concentrations of CTHs for 12h at 37°C. CXCL10 levels were measured by ELISA. (TIFF) [file ppat.1005319.s001.tiff]

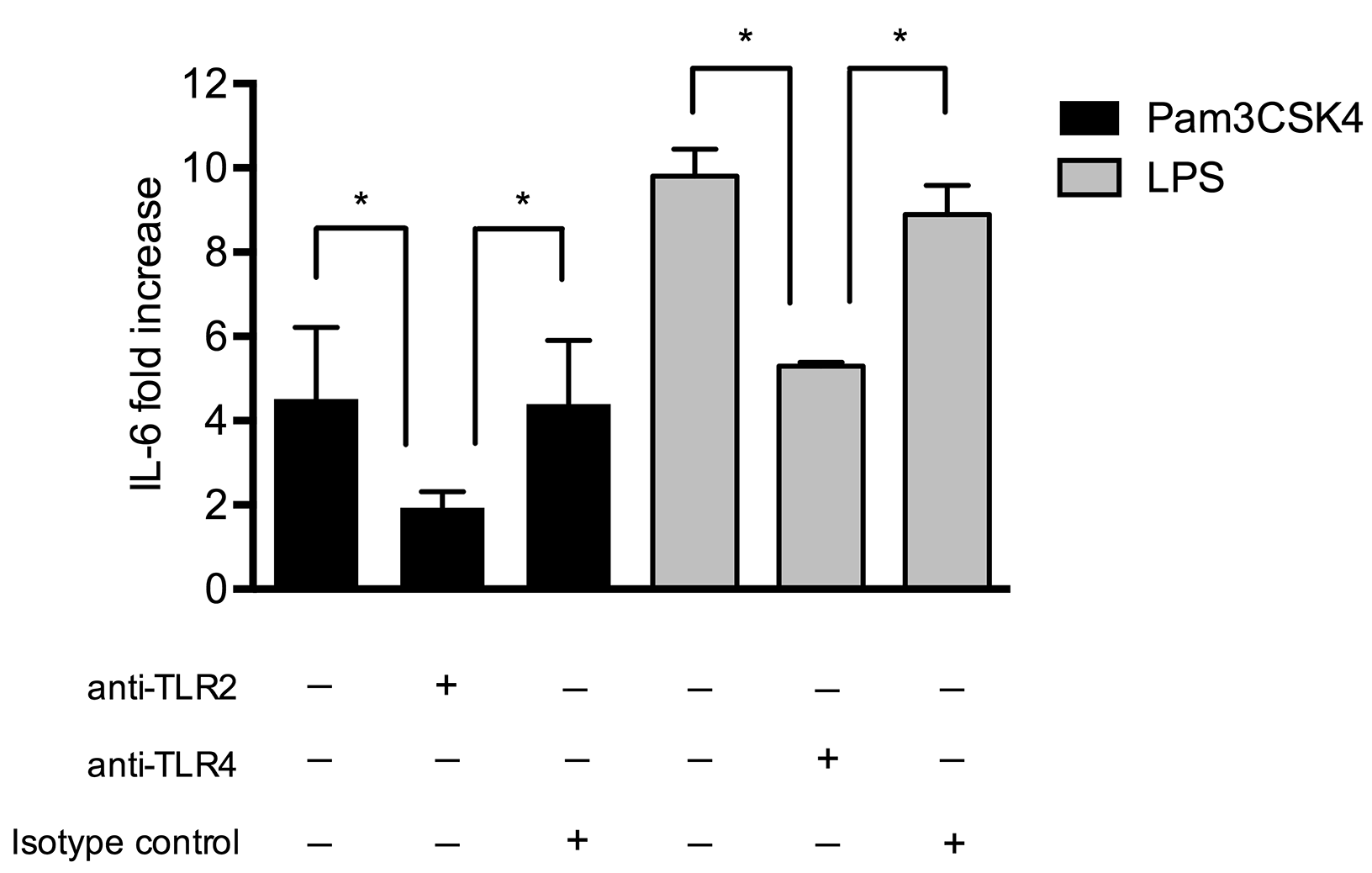

Supplement: S2 Fig — Pam3CSK4 (200 ng/ml) or LPS (100 ng/ml) was added to heparinized blood in presence of antibodies against human TLR2 (25 μg/ml), human TLR4 (25 μg/ml), or an isotype control (25 μg/ml). After a 12-hour incubation at 37°C, the release of IL-6 was measured. Blood stimulated with CTHs in the absence of an antibody served as control. (TIFF) [file ppat.1005319.s002.tiff]

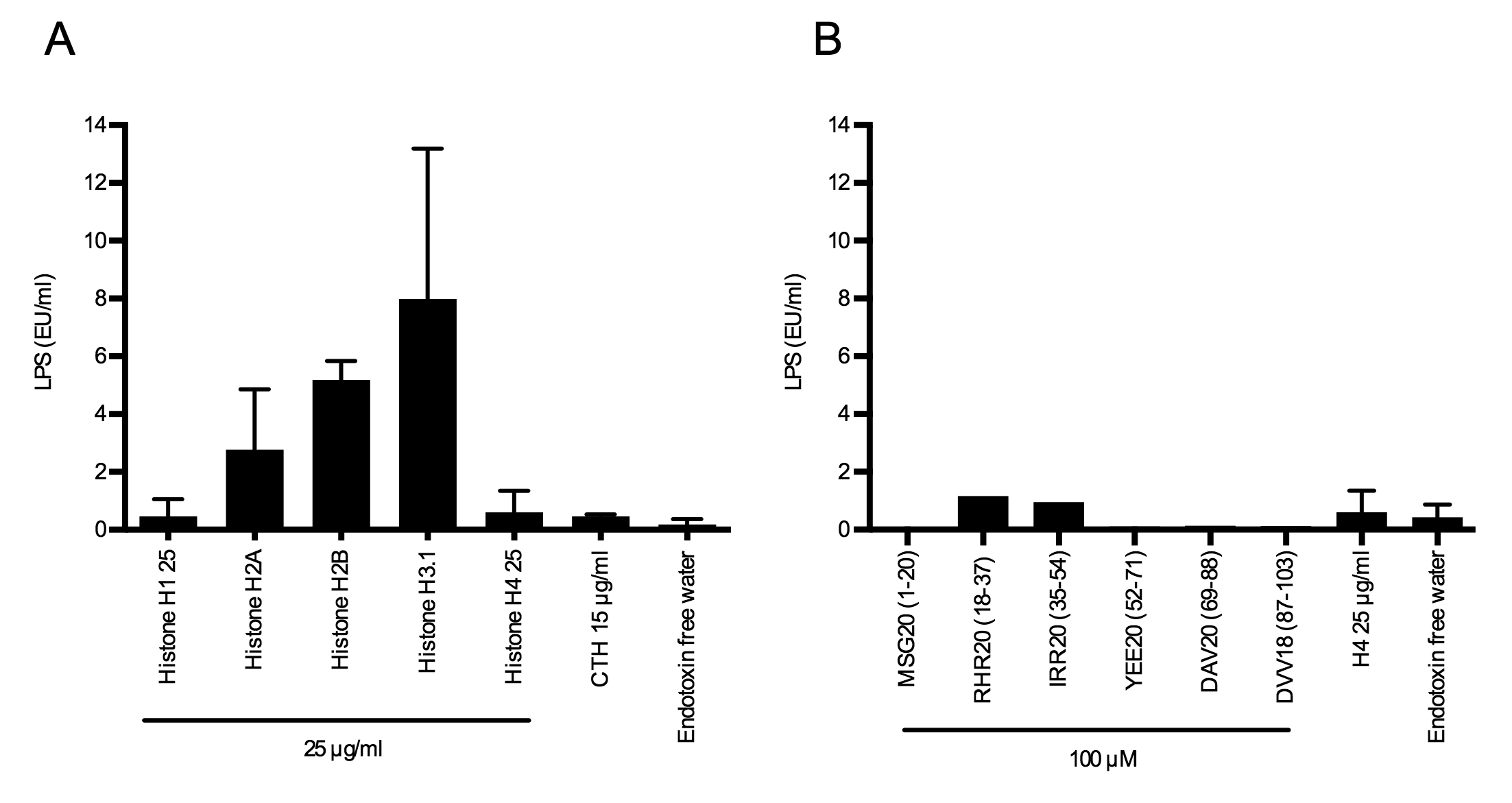

Supplement: S3 Fig — Traces of LPS were measured in different histone subclasses (A) and histone H4-derived synthetic peptides (B) using Pierce LAL Chromogenic Endotoxin Quantitation Kit. (TIFF) [file ppat.1005319.s003.tiff]
